# Regulation of Mouse Small Heat Shock Protein αb-Crystallin Gene by Aryl Hydrocarbon Receptor

**DOI:** 10.1371/journal.pone.0017904

**Published:** 2011-04-11

**Authors:** Shuang Liu, Joram Piatigorsky

**Affiliations:** 1 Laboratory of Molecular and Developmental Biology, National Eye Institute, National Institutes of Health, Bethesda, Maryland, United States of America; 2 Laboratory of Experimental Immunology, National Cancer Institute, National Institutes of Health, Frederick, Maryland, United States of America; University of South Florida College of Medicine, United States of America

## Abstract

The stress-inducible *small heat shock protein (shsp)/αB-crystallin* gene is expressed highly in the lens and moderately in other tissues. Here we provide evidence that it is a target gene of the aryl hydrocarbon receptor (AhR) transcription factor. A sequence (−329/−323, *CATGCG*A) similar to the consensus xenobiotic responsive element (XRE), called here XRE-like, is present in the *α*BE2 region of *αB-crystallin* enhancer and can bind AhR *in vitro* and *in vivo*. αB-crystallin protein levels were reduced in retina, lens, cornea, heart, skeletal muscle and cultured muscle fibroblasts of *AhR^−/−^* mice; αB-crystallin mRNA levels were reduced in the eye, heart and skeletal muscle of *AhR^−/−^* mice. Increased AhR stimulated *αB-crystallin* expression in transfection experiments conducted in conjunction with the aryl hydrocarbon receptor nuclear translocator (ARNT) and decreased AhR reduced *αB-crystallin* expression. AhR effect on *aB-crystallin* promoter activity was cell-dependent in transfection experiments. AhR up-regulated *αB-crystallin* promoter activity in transfected HeLa, NIH3T3 and COS-7 cells in the absence of exogenously added ligand (TCDD), but had no effect on the *αB-crystallin* promoter in C_2_C_12_, CV-1 or Hepa-1 cells with or without TCDD. TCDD enhanced AhR-stimulated *αB-crystallin* promoter activity in transfected *α*TN4 cells. AhR could bind to an XRE-like site in the *αB-crystallin* enhancer *in vitro* and *in vivo*. Finally, site-specific mutagenesis experiments showed that the XRE-like motif was necessary for both basal and maximal AhR-induction of *αB-crystallin* promoter activity. Our data strongly suggest that AhR is a regulator of *αB-crystallin* gene expression and provide new avenues of research for the mechanism of tissue-specific *αB-crystallin* gene regulation under normal and physiologically stressed conditions.

## Introduction

**Figure 1:**
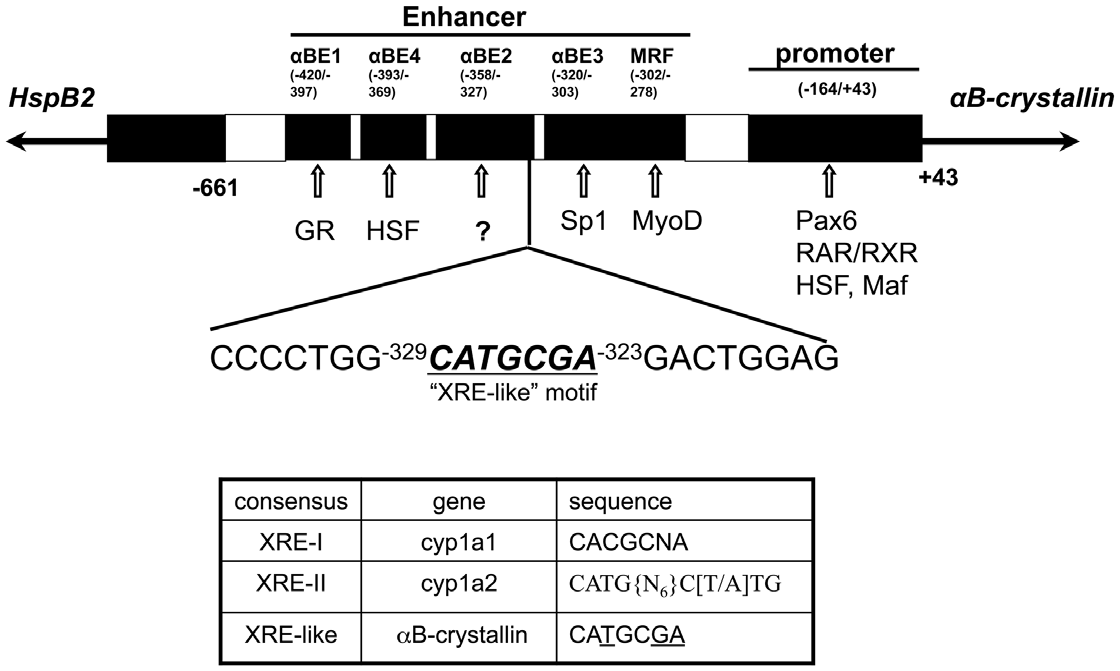
Cis-elements in the intergenic region of mouse *αB-crystallin* and *HspB2* gene and the potential AhR binding site (“XRE-like” motif). Some binding sites for transcriptional factors are located in the indicated elements. There is a potential AhR binding site indicated as “XRE-like” motif identified by bioinformatics. The core sequence (−323/−329) is underlined. GR, Glucocorticoid Receptor; HSF, Heat Shock Factor; RAR, Retinoic Acid Receptor; RXR, Retinoid X Receptor; Maf, Macrophage Activating Factor.

The *αB-crystallin* and *HspB2* genes, both members of the small heat shock protein gene family, are arranged head-to-head with an approximate 1 kb intergenic region in the human, mouse and rat genomes [1]. In the mouse, the intergenic sequence contains an orientation-dependent enhancer that differentially directs expression of the *αB-crystallin* gene to different tissues, with the highest expressions being in the eye lens, heart and skeletal muscle [2], [3]. Although little is known about regulation of *HspB2* gene expression, *αB-crystallin* gene expression has been well studied. Specific transcription factors are known that regulate the proximal promoter and each of the enhancer *cis*-elements, except for the αBE2 enhancer element, of the *αB-crystallin* gene [4], [5], [6], [7], [8], [9] (Fig. 1).

In the present investigation we scanned the *HspB2/αB-crystallin* intergenic regulatory region and identified a candidate aryl hydrocarbon receptor(AhR) binding site, *CATGCG*A, at the edge of αBE2 (Fig. 1). The DNA binding site for AhR is called the xenobiotic responsive element (XRE) or dioxin response element (DRE) [10], [11]. The core consensus sequence for a functional XRE present in enhancers of known AhR-regulated genes (i.e. *cyp1a1*, *cyp1a2*, *cyp1b1*) is CACGCNA. Recently another AhR recognizing sequence, XRE-II, was recognized in the enhancer of *cyp1a2* [12]; XRE-II binds the AhR/aryl hydrocarbon receptor nuclear translocator (ARNT) with the mediation of an unknown protein. The core sequence of XRE-II is identified as CATG{N_6_}C[T/A]TG [12], [13]. Since the potential AhR binding site in the αBE2 of *αB-crystallin* is not a perfect match with either XRE-I or XRE-II, we term this site the XRE-like motif.

AhR belongs to the bHLH /PAS (basic Helix-Loop-Helix/Per Arnt Sim) transcription factor family. Members of this family play critical roles in a broad range of biological functions including regulation of circadian rhythm, neurogenesis, hypoxia response and drug metabolism. A well-established mechanism for AhR function of detoxification starts from its activation by a ligand, usually a dioxin, followed by transport to the cell nucleus, where the AhR forms a heterodimer with ARNT. The AhR/ARNT complex binds to XRE motifs in the regulatory regions of its target genes. Most AhR-regulated genes (*cyp1a1*, *cyp 1a2*, *cyp 1b1*, *glutathione-S-transferase Ya* and *NAD(P)H-quinone oxidoreductase*) metabolize xenobiotics [14], [15], [16]. While the detoxification role of AhR has been well investigated, very little is known about the other functions of this ancient protein. Recent studies on AhR null mice have revealed *de novo* physiologic functions of AhR in addition to induction of detoxification genes, and many new potential AhR regulated genes have been identified by high-throughput technologies [13], [17], [18]. The present study provides evidence that AhR regulates *small heat shock protein*/*αB-crystallin* gene expression in a ligand-independent fashion.

## Results

### αB-Crystallin is Decreased in AhR Null Mice

**Figure 2:**
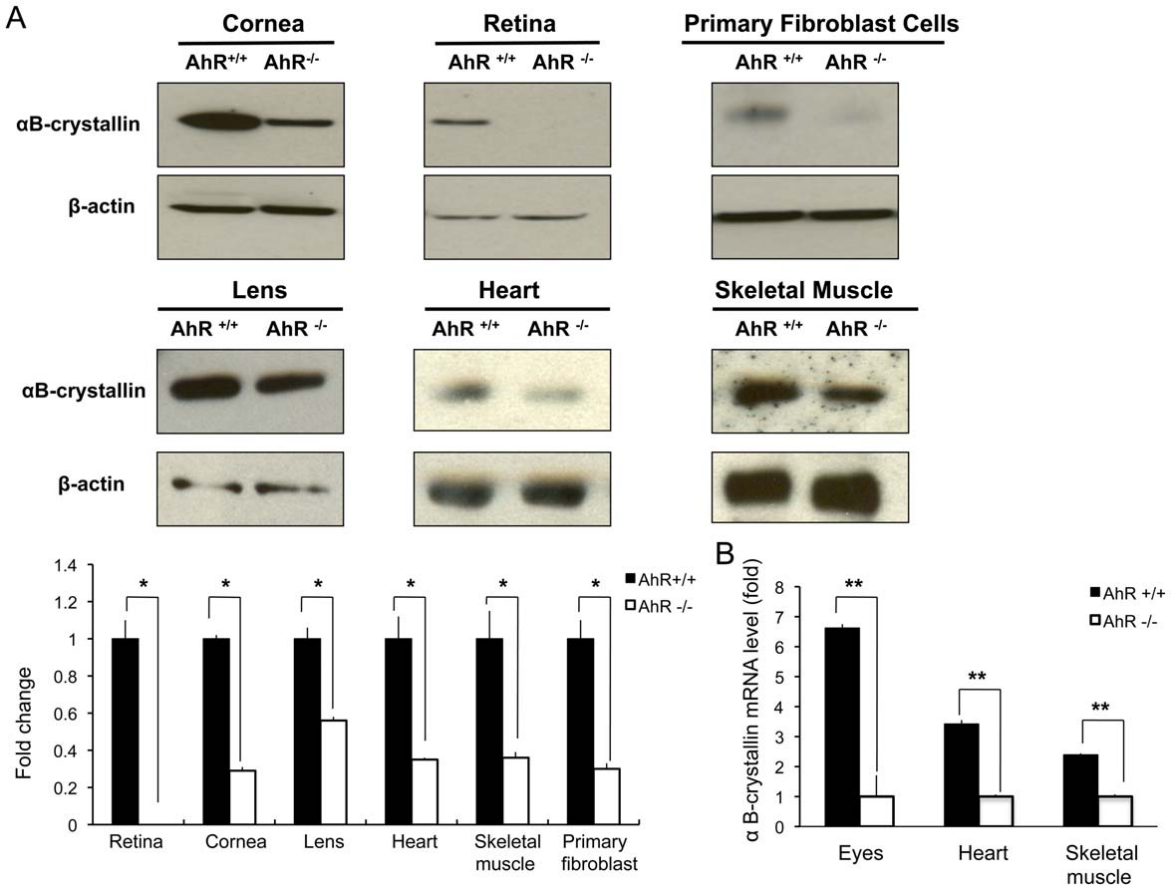
αB-crystallin is decreased in AhR^−/−^ mice. A. Cornea, retina, lens, heart, and skeletal muscle were isolated from adult AhR^−/−^ and AhR^+/+^ mice and tissue proteins extracted. Primary fibroblasts from skeletal muscle were cultured and cell proteins extracted. αB-crystallin was analyzed by Western immunoblotting. β-actin was detected as an internal control. (n = 4, *, p<0.05). B. αB-crystallin mRNA levels were determined by real time-PCR using total RNA extracted from whole eyes, heart and limb skeletal muscle. GAPDH mRNA levels were determined as an internal control (n = 2, **, p<0.01).

We examined *αB-crystallin* expression levels in different tissues of adult *AhR^−/−^* and *AhR^+/+^* mice [19]. αB-crystallin protein levels in the retina, lens, cornea, heart and skeletal muscles were reduced in AhR^−/−^ mice compared to AhR^+/+^ mice (Fig. 2A).

The retina, lens, cornea, heart and skeletal muscle contain either abundant (lens, heart, skeletal muscle) or moderate (cornea, retina) levels of αB-crystallin [20], [21], [22], [23]. αB-crystallin was undetectable in the retina and reduced by approximately 3-fold in the cornea, heart and skeletal muscle, and 2-fold in the lens of AhR^−/−^ mice. β-actin was at comparable levels in these tissues in the AhR^−/−^ and AhR^+/+^ mice. αB-crystallin mRNA levels were reduced approximately 7-fold in the eyes (whole eyeball), 3-fold in the heart and 2-fold in skeletal muscle of the AhR^−/−^ mice compared to the age-matched AhR^+/+^ mice (Fig. 2B).

We also compared the levels of αB-crystallin in primary cultures of muscle derived fibroblast cells from AhR^−/−^ and AhR^+/+^ mice (Fig. 2A). There was much less ( 0.3-fold) αB-crystallin in AhR^−/−^ fibroblasts compared to AhR^+/+^ fibroblasts. The absence of AhR in the primary AhR^−/−^ fibroblast cells was confirmed by immunoblotting (data not shown).

**Figure 3:**
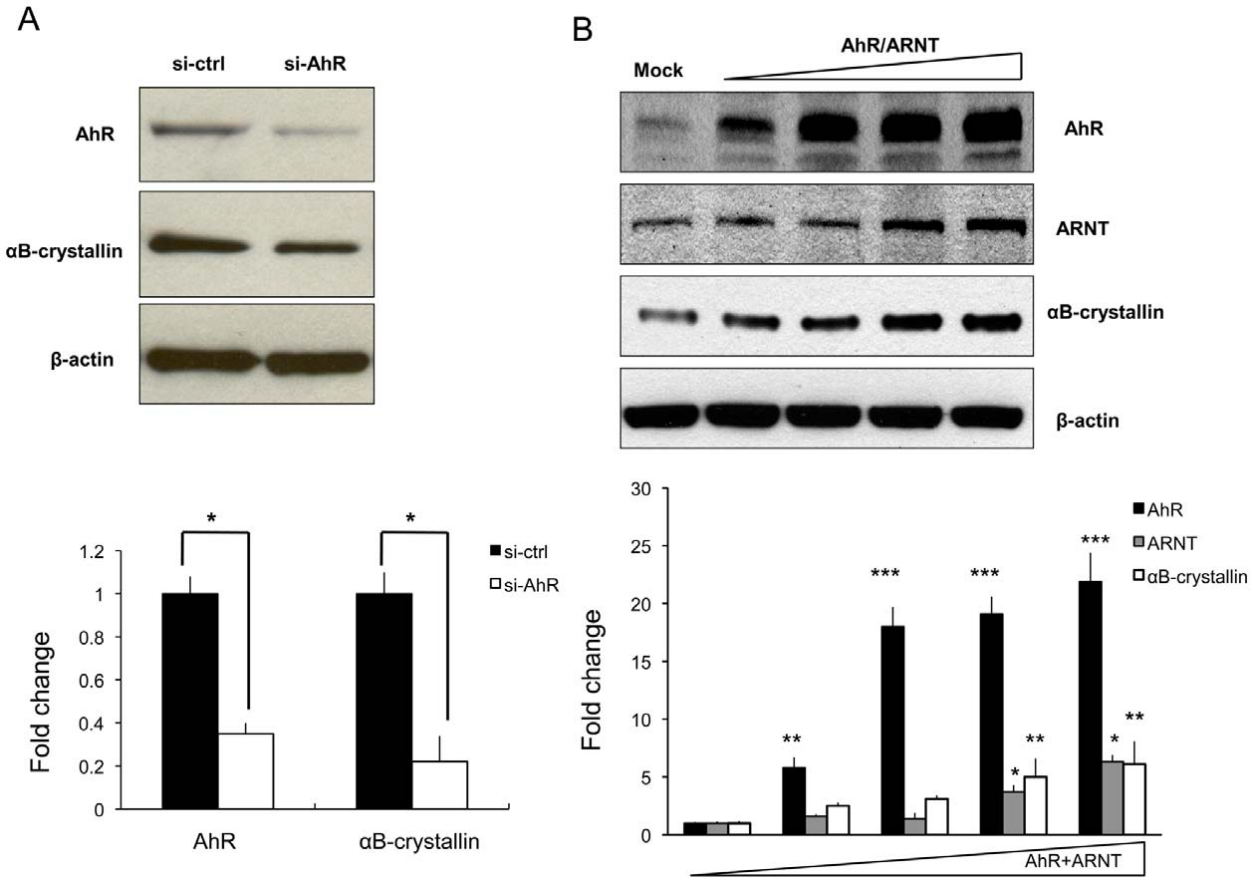
The effects of AhR knock-down and over-expression on αB-crystallin expression. A. 80 pmol of siRNA targeting AhR mRNA (si-AhR) or non-targeting siRNA (si-ctrl) were transfected into αTN4 cells and total cell proteins were extracted 60 h later. Expression of AhR and αB-crystallin were determined by Western immunoblotting. B. 0.1, 0.25, 0.5 and 1 µg pcDNA3.1/B6AhR and pcDNA/ARNT were co-transfected into αTN4 cells and total cell proteins were extracted 24 h later. AhR, ARNT and αB-crystallin were detected by Western immunoblotting. β-actin was detected as an internal control. ( *, p<0.05; **, p<0.01; ***, p<0.001).

We next tested whether reducing AhR levels by siRNA or increasing AhR levels using plasmids encoding AhR and ARNT would affect αB-crystallin levels in cultured rabbit lens αTN4 cells. AhR siRNA reduced AhR level by 65% and αB-crystallin level by 78%, respectively (Fig. 3A). By contrast, overexpression of AhR/ARNT increased αB-crystallin expression in a dose dependent manner (Fig. 3B). Together, these results are consistent with AhR being a positive regulator of *αB-crystallin* gene expression.

### AhR/ARNT Upregulates αB-crystallin Promoter Activity Independently of Exogenous Ligand

**Figure 4:**
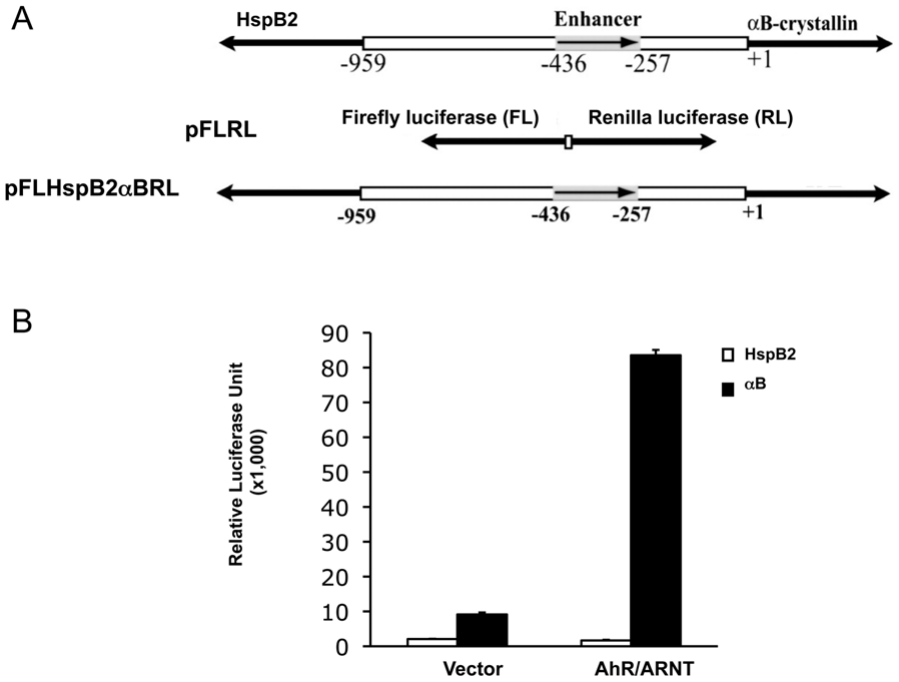
The effects of AhR/ARNT on *αB-crystallin* promoter activity. A. Structure of the pFLHspB2αBRL dual reporter plasmid used for reporter assays. B. AhR/ARNT up-regulates *αB-crystallin* but not *HspB2* promoter activity. 50 ng pcDNA3.1/B6AhR and 50 ng pcDNA/ARNT were co-transfected into HeLa cells with 100 ng pFLHspB2αBRL and 10 ng β-gal control vector. Luciferase activities were determined 48 h later. Data are presented as the ratio of firefly luciferase activity to β-gal activity (Relative Luciferase Unit, RLU) to indicate *HspB2* promoter activity, or the ratio of *Renilla* luciferase activity to β-gal activity to indicate *αB-crystallin* promoter activity. The results are presented as mean values; S.D. values were derived from three independent experiments.

**Figure 5:**
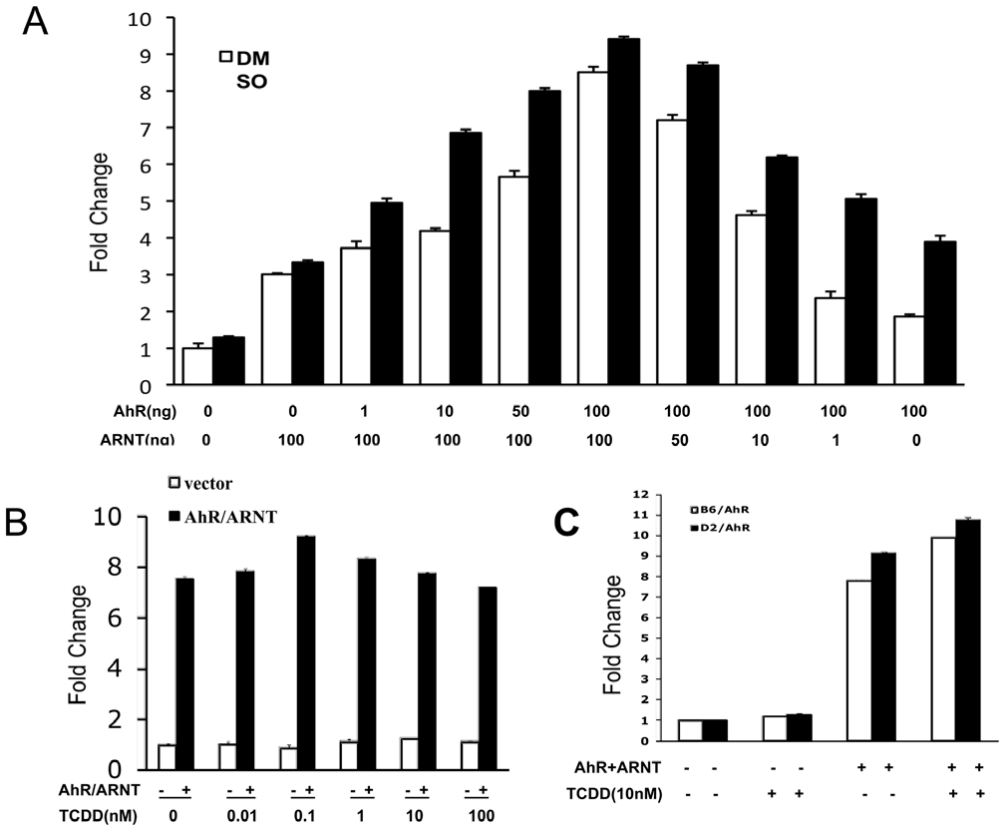
AhR/ARNT up-regulates *αB-crystallin* promoter activity independently of TCDD. A. AhR and ARNT dose-dependently up-regulate *αB-crystallin* promoter activity. Increasing doses of pcDNA3.1/B6AhR and pcDNA/ARNT were co-transfected with fixed amounts of pFLHspB2αBRL and β-gal control vector into HeLa cells. After 24 h the cells were treated for another 24 h with TCDD (10 nM) or DMSO (0.01%) and then assayed for luciferase activities. B. Effects of TCDD on *αB-crystallin* promotor activity. HeLa cells transfected with pcDNA3.1/B6AhR and pcDNA/ARNT or control vector were treated with progressive concentrations of TCDD as indicated. After 24 h the cells were assayed for luciferase activities. **C**. Effects of high ligand-affinity AhR (B6/AhR) and low ligand-affinity AhR (D2/AhR) on *αB-crystallin* promoter activity. HeLa cells were co-transfected with pcDNA3.1/B6AhR or pcDNA3.1/D2AhR with pFLHspB2αBRL and β-gal control vector. After 24 h the cells were treated with TCDD (10 nM) or DMSO (0.01%) for another 24 h and then assayed for luciferase activities. The fold change was recorded by determining luciferase activity in AhR/ARNT transfected and/or TCDD-treated cells relative to that in the pcDNA3.1 transfected and DMSO-treated cells. The means and S.D. values were derived from three independent experiments.

We next examined whether AhR can regulate *αB-crystallin* promoter activity. We used a duel reporter plasmid, pFLHspB2αBRL [3], which contains the 1 kb intergenic regulatory region between the head-to-head *αB-crystallin* and *HspB2* genes. In this plasmid the proximal promoter of *HspB2* drives the firefly luciferase gene and the proximal promoter of *αB-crystallin* drives the *Renilla* luciferase gene (Fig. 4A), thus allowing us to detect the effect of transcription factors on each of the promoters simultaneously. HeLa cells were transfected with the plasmids encoding mouse AhR and ARNT and the *Renilla* and firefly luciferase activities were determined. As shown in Fig. 4B, AhR/ARNT had no significant effect on *HspB2* promoter activity, but increased *αB-crystallin* promoter activity by 9.1-fold. In order to identify individual contributions of AhR and ARNT, we tested their dose effects individually on *αB-crystallin* promoter activity. AhR and ARNT each up-regulated *αB-crystallin* promoter activity in a dose-dependent fashion; the combination of AhR and ARNT had an additive positive effect on the activity of the *αB-crystallin* promoter (Fig. 5A). By contrast, AhR/ARNT had little, if any, effect on *HspB2* promoter activity (data not shown).

Unexpectedly, our data indicated that AhR does not require an exogenous ligand to stimulate *αB-crystallin* promoter activity. Thus, we further tested the effect of 2,3,7,8-tetrachlorodibenzo-p-dioxin (TCDD), the prototypical ligand of AhR [15], [16], [24], on the ability of AhR to stimulate *αB-crystallin* promoter activity. Fig. 5B shows that TCDD did not augment the stimulation of *αB-crystallin* promoter activity by AhR/ARNT. In additional tests, a high ligand-affinity AhR (C57BL/6AhR) and a low ligand-affinity AhR (DBA/2AhR) [25] up-regulated *αB-crystallin* promoter activity by 7.8-fold and 9.2-fold, respectively, in the absence of TCDD (Fig. 5C), confirming the ligand-independence of AhR stimulation of *αB-crystallin* promoter activity.

**Figure 6:**
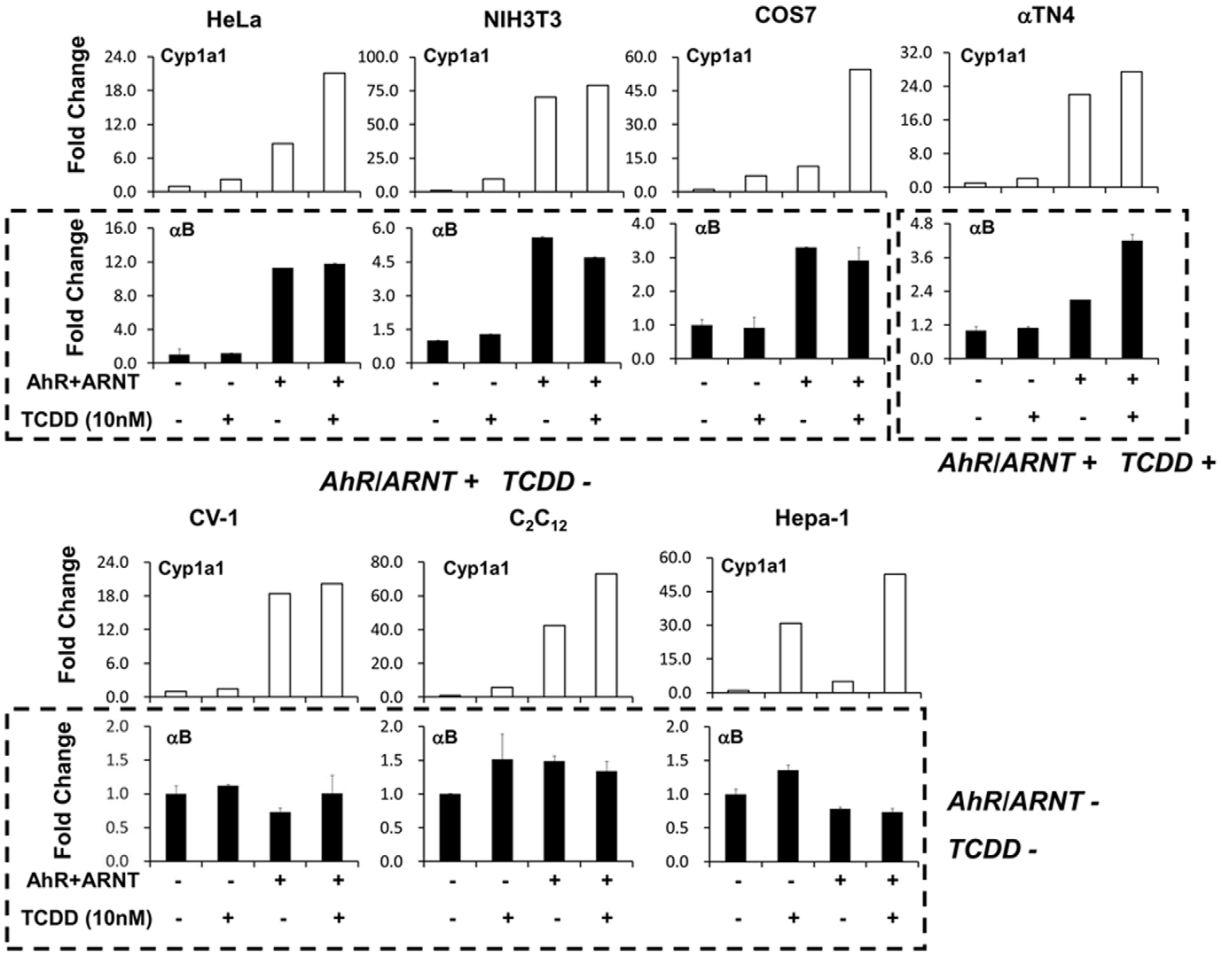
AhR/ARNT up-regulates *αB-crystallin* promoter activity in a cell-type dependent manner. pFLHspB2αBRL or the cyp1a1 reporter plasmid was co-transfected with pcDNA3.1/B6AhR, pcDNA/ARNT and β-gal control vector into the specified cells as indicated and the cells were cultured for 24 h. Cells were incubated with TCDD (10 nM) or DMSO (0.01%) for another 24 h before luciferase activities were determined. The fold change was recorded by determining luciferase activity in AhR/ARNT transfected and/or TCDD-treated cells relative to that in the pcDNA3.1 transfected and DMSO-treated cells. The means and S.D. values were derived from three independent experiments. Three types of AhR/ARNT and TCDD induced-*αB-crystallin* promoter responses were detected, depending on the cell type, as indicated under the boxed results. AhR/ARNT increased *Cyp1a1* promoter activity in all the cell types tested and showed variable, cell-type dependent additional stimulation in the presence of TCDD.

It has been reported that AhR activity differs among cell types [24], [25], [26]. Thus we examined the AhR/ARNT regulation on *αB-crystallin* promoter in seven different cell lines and used the *cyp1a1* promoter, a target gene positively regulated by AhR/ARNT [10], [11], as a positive control. Interestingly, the results differed with cell type (Fig. 6). In HeLa, NIH3T3 and COS-7 cells, AhR/ARNT increased *αB-crystallin* promoter activity by 11.3-fold, 5.5-fold and 3.3-fold, respectively, in a TCDD-independent manner; in CV-1, C_2_C_12_ and Hepa-1 cells, AhR/ARNT did not stimulate *αB-crystallin* promoter activity; in αTN4 lens cells, AhR/ARNT increased *αB-crystallin* promoter activity by 2.1-fold, and this stimulation was increased to 4.2-fold by the addition of TCDD. As expected, AhR/ARNT increased *cyp1a1* promoter activity in all the cell types tested; AhR/ARNT stimulation was appreciably increased by the addition of TCDD in HeLa, COS-7 and Hepa-1 cells, moderately enhanced in C_2_C_12_ cells, and slightly elevated if at all in NIH3T3 and CV-1 cells (Fig. 6). We conclude from these experiments that AhR/ARNT can positively regulate *αB-crystallin* promoter activity TCDD independently or dependently depending upon the cell type.

### AhR Binds the XRE-like Motif in the αB-Crystallin 5′-Regulatory Region

**Figure 7:**
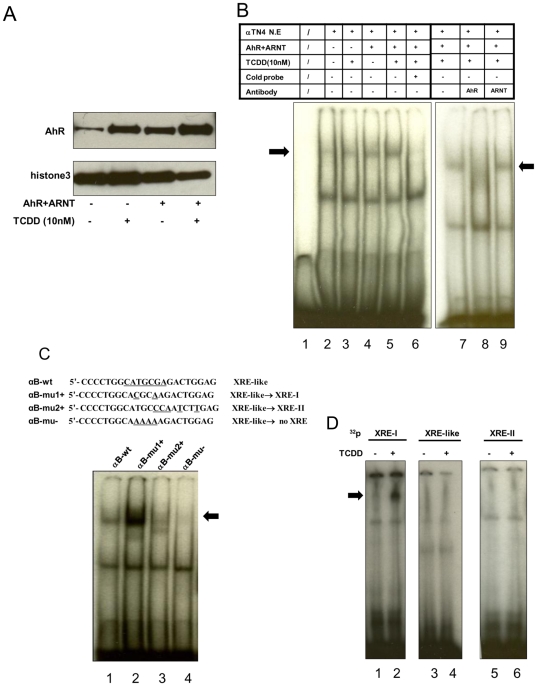
AhR binds to the XRE-like motif in the *αB-crystallin* enhancer. A. Nuclear AhR levels in αTN4 cells. αTN4 cells were transfected with pcDNA3.1/B6AhR and pcDNA/ARNT or pcDNA3.1 for 24 h, treated with or without 10 nM TCDD for 2 h, and then nuclear extracts were prepared. AhR protein levels were tested by western immunoblotting. Histone 3 was immunoblotted as an internal control. B. The −336/−315 fragment of the mouse *αB-crystallin* gene containing the XRE-like motif was synthesized and labeled with ^32^P. Nuclear extracts were prepared from αTN4 cells transfected with pcDNA3.1/B6AhR and pcDNA/ARNT, treated with 10 nM TCDD or DMSO (0.01%) for 2 h. Gel shift was performed as described under “ Materials and Methods”. Arrows indicate the specific protein-DNA band. The band was induced by AhR/ARNT and increased by the combination of AhR/ARNT and 10 nM TCDD. The band was competed by 25-fold excess unlabeled probe or AhR antibody, but not by ARNT antibody. C. Gel shifts testing the binding of AhR to the XRE-like motif or to its mutant sequences. Equal amounts of nuclear extract from αTN4 cells transfected with pcDNA3.1/B6AhR and pcDNA/ARNT, treated with TCDD were incubated with a ^32^P-labeled XRE-like sequence or with mutated ^32^P-labeled XRE-I, XRE-II or non-XRE sequences, as indicated; the protein-DNA complexes were analyzed by gel shift. Arrows indicate the specific bands. D. *In vitro* translated AhR/ARNT did not bind to the XRE-like motif. Mouse AhR and ARNT were synthesized in a rabbit reticulocyte lysate in the presence of 100 nM TCDD or vehicle for 90 min. The binding capability of the *in vitro* synthesized AhR/ARNT to the XRE-like, XRE-I and XRE-II motifs were tested by gel shift.

Gel shift and ChIP assays were performed to test whether AhR can bind the XRE-like motif that we identified in the *αB-crystallin* enhancer. In gel shift experiments using αTN4 cell nuclear extracts and a 22 bp oligonucleotide containing the XRE-like motif (indicated in Fig. 7C as αB-wt), a weak but specific band representing the AhR/XRE-like motif complex was detected in untreated αTN4 cells (Fig. 7B, lane 2). AhR/ARNT transfection and TCDD addition increased the band intensity individually (Fig. 7B, lane 3, 4) and additively when combined (Fig. 7B, lane 5). Western blot also revealed separate and additive effects of AhR/ARNT transfection and TCDD addition on nuclear AhR accumulation (Fig. 7A). The band was competed by a 25-fold molar excess of non-radioactive probe (Fig. 7B, lane 6), and became smeared or decreased in intensity by the addition of anti-AhR antibody to the reaction mixture (Fig. 7B, lane 8). The anti-ARNT antibody did not affect the bands (Fig. 7B, lane 9). In further gel shift experiment using HeLa cells (data not shown), the specific band of complex was more intense using nuclear extracts from cells transfected with the AhR/ARNT vector than from cells transfected with the control vector (pcDNA3.1). Not as in the experiments with the αTN4 cells, TCDD did not increase the band intensity in the experiments with HeLa cells. These results are consistent with our functional tests using αTN4 and HeLa cells indicating that AhR regulates the *αB-crystallin* promoter (see Fig. 6).

We next examined the binding specificity of AhR to the XRE-like motif ( *CATGCG*A) by mutating this site (αB-wt) to an XRE-I (CACGCAA) sequence (αB-mu1+), an XRE-II (CATGCCCAATCTT) sequence (αB-mu2+), or a sequence lacking an XRE site (αB-mu−; CAAAAAA) (Fig. 7C). Protein-oligonucleotide complex formation was more intense with the αB-mu1+ sequence (Fig. 7C, lane 2) and weaker with the aB-mu2+ sequence (lane 3) than with the αB-wt sequence (Fig. 7C, lane 1). No discrete band was produced with the αB-mu− (Fig. 7C, lane 4). These results are consistent with DNA sequence-specific binding of AhR to XRE-related motifs.

A previous report indicated that *in vitro* translated AhR/ARNT does not bind to the XRE-II motif, suggesting that *in vivo* binding requires additional proteins or posttranslational modification(s) [12]. Therefore, we examined the capacity of *in vi*tro translated AhR/ARNT to bind the XRE-like motif (Fig. 7D). The AhR/ARNT generated by a rabbit reticulocyte expression system bound the XRE-I motif in the presence of TCDD (100 nM) (Fig. 7D, lane 2) but did not bind the XRE-like motif in the gel shift experiments (Fig. 7D, lane 3,4). Either binding of the *in vitro* generated AhR/ARNT to the XRE-like and/or XRE-II sequences required modification that was not performed by the system, or other factor(s) are needed for binding to occur.

**Figure 8:**
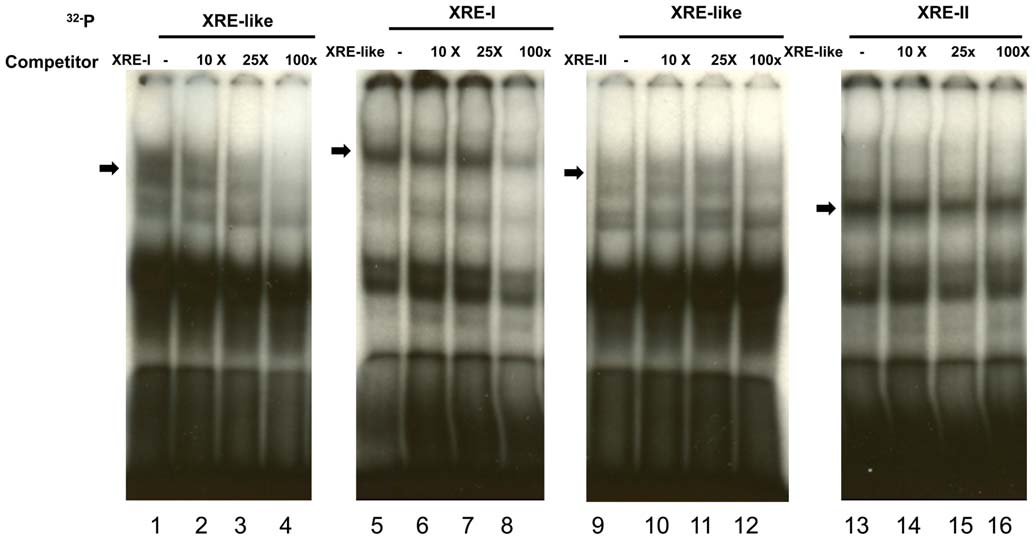
Competitions between XRE-like motif and XRE-I motif or XRE-II motif for AhR binding. The XRE-like, XRE-I and XRE-II motif oligonuccleotides were labels with ^32^P and incubated with nuclear extracts from pcDNA3.1/B6AhR and pcDNA/ARNT transfected, TCDD (10 nM)-treated HeLa cells. 10-, 25-, and 100-fold molar excess of unlabeled oligonucleotides were pre-incubated with the nuclear extract prior to incubation with the labeled oligonucleotides. The bands indicated by arrows in the gel shift experiments represent the specific complexes of AhR.

The binding abilities of AhR to the XRE-like, XRE-I and XRE-II motifs were compared by competition experiments using HeLa cells (Fig. 8). A 10, 25, and 100-fold molar excess of XRE-I motif gradually eliminated the binding of AhR to the XRE-like motif (Fig. 8, Lane 1–4); by contrast, appreciable complex of AhR to the XRE-I sequence remained even at a 100-fold molar excess of the XRE-like sequence (Fig. 8, lanes 5–8), consistent with stronger AhR binding to XRE-I than to the XRE-like sequence. There was no competition for AhR binding between the XRE-like and the XRE-II motifs (Fig. 8, lanes 9–16).

**Figure 9:**
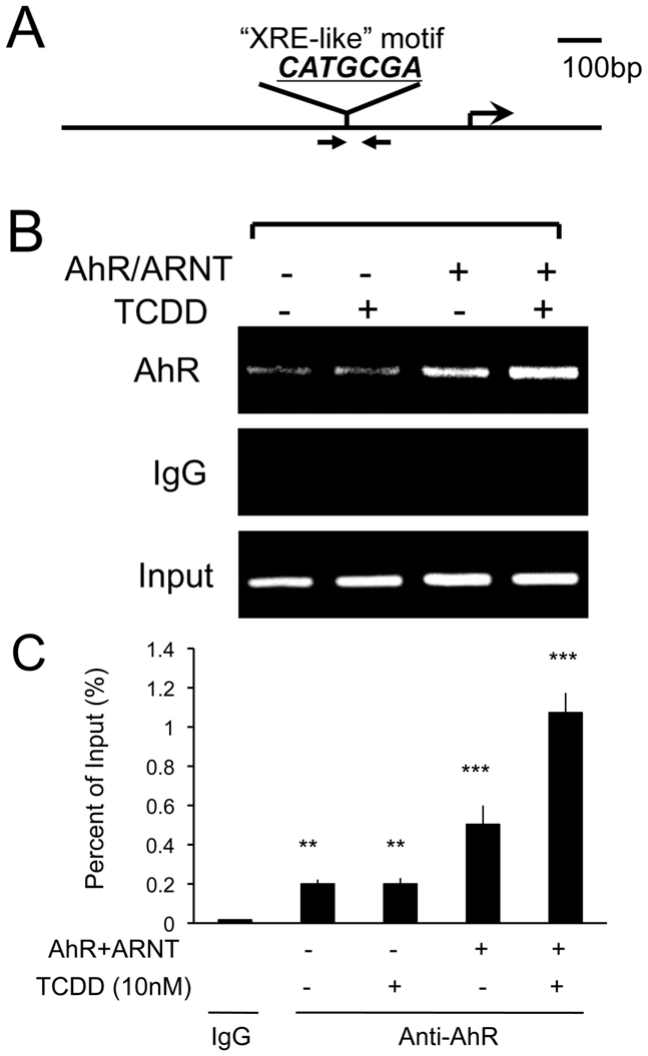
AhR binds *in vivo* to *αB-crystallin* enhancer containing the XRE-like motif. αTN4 cells were transfected with pcDNA3.1/B6AhR and pcDNA/ARNT and treated with TCDD (10 nM) for 2 h. Cells were subjected to ChIP assay described under “Materials and Methods”. A. Schematics of the regions in αB-cystallin enhancer for amplification by ChIP. Arrows indicate the locations of the primers. B. C. The AhR-associated DNA was immunoprecipitated with anti-AhR antibody and amplified by PCR (B) and real time PCR (C). A specific band of 250 bp could be amplified only from which contained the XRE-like site. The samples that were precipitated with IgG did not give any amplified product. The specificity of the commercially obtained antibody has been confirmed by Western immunoblotting elsewhere [40]. (**, p<0.01; ***, p<0.001).

We next tested the *in vivo* interaction of AhR with the XRE-like motif in αTN4 cells by ChIP analysis (Fig. 9). DNA isolated from anti-AhR antibody precipitated complexes was amplified by PCR and real time-PCR using a pair of primers that could amplify the sequence containing the XRE-like motif (Fig. 9A). The predicted band of 250 bp was detected in untransfected αTN4 cells indicating complex formation in the untreated cells. In AhR/ARNT transfected αTN4 cells, a much stronger band was detected and TCDD addition further increased the band intensity (Fig. 9B, C). The results of these ChIP experiments are consistent with our functional transfection and gel mobility shift experiments suggesting an *in vivo* role for AhR in αB-crystallin gene expression.

### The XRE-like Motif is Necessary for Basal αB-Crystallin Promoter Activity and Maximal AhR/ARNT-Inducible αB-Crystallin Promoter Activity

**Figure 10:**
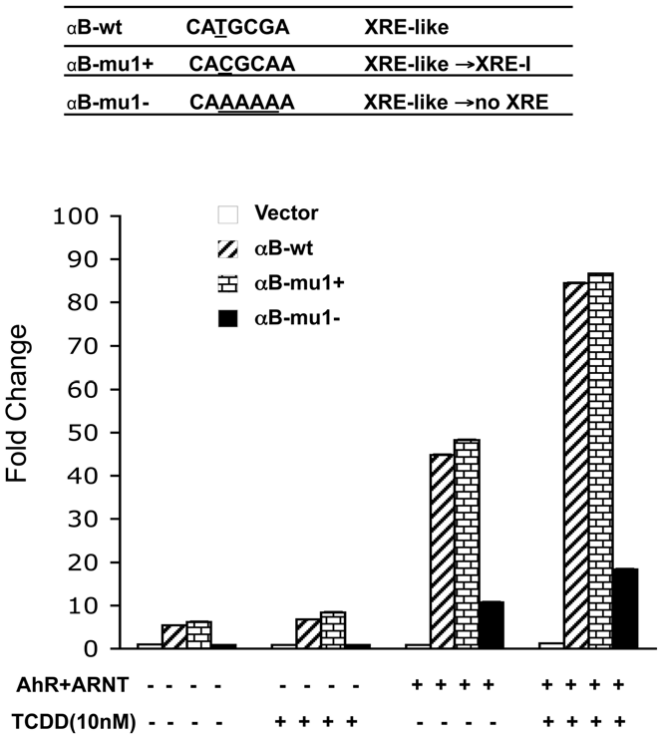
The XRE-like motif is necessary for basal and maximal AhR/ARNT induced-*αB-crystallin* promoter activity. Mutations were introduced in the XRE-like motif by site-directed mutagenesis as shown by the underlined bases. HeLa cells were co-transfected with the indicated luciferase constructs, pcDNA3.1/B6AhR and pcDNA/ARNT or pcDNA3.1. After 24 h the cells were treated with TCDD (10 nM) or DMSO (0.01%) for another 24 h and luciferase activities were determined. The fold change was recorded as the luciferase activity in the cells transfected with the AhR and ARNT constructs relative to that in the cells transfected with the empty vector (pGL3-basic). The means and S.D. values were derived from three independent experiments.

We tested the functional importance of the XRE-like motif for *αB-crystallin* promoter activity. The XRE-like motif in the reporter construct was mutated either to an XRE-I sequence or to a non-XRE sequence identical to those used for the binding assays above (Fig. 10). Mutating the XRE-like motif to a non-XRE sequence reduced basal promoter activity by 85% and reduced maximal AhR/ARNT-induced promoter activity by 86% relative to the wild type *αB-crystallin* promoter activity (Fig. 10). Thus the XRE-like motif is necessary and important for basal *αB-crystallin* promoter activity and maximal AhR/ARNT-inducibility.

## Discussion

The present report suggests that *αB-crystallin* is a target gene for the transcription factor, AhR. αB-crystallin protein expression was reduced in the eyes (lens and cornea), heart, skeletal muscle and primarily cultured fibroblasts in AhR^−/−^ mice. The AhR^−/−^ mice had no detectable αB-crystallin in the retina, where both αB-crystallin and AhR are known to be prevalent in vertebrates and invertebrates [22], [23], [27], [28], [29], [30]. Although no connection is known between αB-crystallin and retinal function, it is noteworthy that disruption of the *Drosophila* AhR ortholog, *Spineless*, disables color vision in this species [29].

The present data indicate that αB-crystallin protein and mRNA levels are decreased in the eyes of AhR^−/−^ mice; however, histological examination of these eyes revealed no obvious morphological abnormality in the eyes of either adult or 19 day-old postnatal pups of AhR^−/−^ mice (data not shown). In this connection, it is noteworthy and consistent with reports that although AhR protein [31], [32] and αB-crystallin mRNA [33] are highly expressed in the lens of 12–15 day-old mouse embryos, the lenses of neither AhR null mice [34] nor αB-crystallin [35] null mice appear abnormal. Thus, the precise developmental roles of AhR and αB-crystallin in the lens, as well as in other tissues, require additional investigation.

Our strongest evidence that AhR regulates *αB-crystallin* gene expression comes from the combination of transfection experiments that tested for promoter function and gel mobility shift assays that tested for direct binding to DNA. AhR stimulated the *αB-crystallin* promoter in transfected cells and formed a complex with the XRE-like site in the *αB-crystallin* enhancer. Mutation of this site reduced both promoter activation and AhR binding. Mutation of the XRE-like site also lowered the basal promoter activity of the *αB-crystallin* gene as well as the ability of the promoter to be activated by AhR. It is noteworthy, however, that AhR did not stimulate *αB-crystallin* promoter activity in all types of transfected cells. Positive results were obtained in HeLa, NIH3T3, COS-7 and αTN4 cells, but not in C_2_C_12_, CV-1 or Hepa-1 cells. The reason for this cell type-dependency is not known, but suggests that specific interaction with one or more factors is essential. It is also intriguing that although the XRE-like site is necessary for both basal promoter activity and AhR-stimulation of promoter activity, it binds AhR weakly. One possibility for the weak binding in our tests is that additional proteins that were not present in the gel shift reaction mixture are required for maximal complex formation. The inability of *in vitro* translated AhR/ARNT to form a complex with the XRE-like site is consistent with this possibility. Further studies on AhR binding to the αB-crystallin promoter both in vivo and in vitro using physiological levels of nuclear proteins are warranted.

Our *in vitro* functional and gel shift experiments, as well as ChIP experiments, indicate that AhR can stimulate *αB-crystallin* promoter activity in the absence of TCDD. A caveat is that TCDD increase of AhR stimulation of the *αB-crystallin* promoter depends on cell type (i.e. αTN4 cells). AhR can also regulate other gene expression, for example *TGFβ1*, independently of TCDD [36]. Indeed, the expression of a large number of genes not affected by TCDD in different tissues was altered in AhR^−/−^ mice [17], [18]. It is unknown whether these represent direct or indirect effects of AhR. The genes altered in the AhR null mice are enriched for classic XRE sites, and it is likely that various transcription factors collaborate with AhR in a combinatorial fashion to regulate tissue-specific mRNA regulation in wild type mice [18]. It remains possible, of course, that a yet unknown endogenous ligand substitutes for TCDD in certain cases or under specific conditions. FICZ (6-formylindolo[3,2-b]carbazole) and/or other tryptophan derivatives are candidate endogenous ligands for AhR [37].

Finally it is noteworthy that in preliminary tests (data not shown) AhR/ARNT increased the activity of a truncated *αB-crystallin* promoter lacking the XRE-like site (−257 to +43) up to 24% of the AhR/ARNT-induced wild type promoter. Since the identified XRE-like site is the only sequence resembling a classical XRE sequence in the 5′ flanking region of the *αB-crystallin* gene, it is possible that AhR stimulated *αB-crystallin* activity of the truncated promoter by binding another sequence *via* one or more scaffolding proteins. Alternatively, AhR/ARNT may have activated another gene that in turn stimulated activity of the truncated promoter. Further studies are necessary to resolve the multiple interactions involved in *αB-crystallin* gene expression.

In summary, whatever the mechanism, our data suggest that AhR/ARNT participates in the regulation of the *αB-crystallin* gene. Apart from its intrinsic interest with respect to the complex mechanism of *aB-crystallin* gene expresssion [38], [39], AhR regulation of the *αB-crystallin* gene is biologically logical: both αB-crystallin and AhR function in mediating cellular stress responses [39], [40].

## Materials and Methods

### Chemicals and Antibodies

2,3,7,8-tetrachlorodibenzo-p-dioxin (TCDD) was purchased from Cambridge Isotope Laboratories, Inc. Anti-AhR antibody (RPT9) and anti-ARNT antibody (2B10) were purchased from Abcam Inc. Anti-αB-crystallin antibody was purchased from Stressgen. Anti-β-actin antibody was purchased from Sigma. Anti-Histone H3 antibody was purchased from Abcam Inc.

*Cell Lines*-HeLa (CCL-2) cells, Hepa1-6 (CRL-1830) cells, C_2_C_12_ (CRL-1772) cells, NIH3T3(CRL-1658) cells, CV-1 (CCL-70) and COS-7 (CRL-1651) cells were purchased from ATCC and maintained in Dulbecco's modified Eagle's medium (DMEM) with 10% fetal bovine serum. αTN4 cells were maintained as previously described [8].

### Plasmids and Site-Directed Mutagenesis

The plasmids encoding mouse AhR protein (pcDNA3.1/B6AhR and pcDNA3.1/D2AHR) and the cyp1a1 reporter plasmid (P1646luc3) were kindly provided by Dr. Alvaro Puga (University of Cincinnati College of Medicine). The plasmid encoding mouse ARNT protein (pcDNA/ARNT) was kindly provided by Dr. Oliver Hankinson (University of California, Los Angeles, California). The dual-directional reporter plasmid pFLHspB2αBRL (in which RL is *Renilla* luciferase and FL is firefly luciferase) is described elsewhere [3]. Site-directed mutagenesis was performed with the QuikChange Site-Directed Mutagenesis kit (Stratagene). The primers containing mutated site were synthesized by Integrated DNA Technologies Inc as follows: αB-mu1+: 5′-TCA ATT CCC CTG GCA CGC AAG ACT GGA GAG (in which CATGCGA→CACGCAA); αB-mu1-: 5′-GGC TCA ATT CCC CTG GCA AAA AA G ACT GGA GAG GAG GAG GGG (in which CATGCGA→CAAAAAA).

*siRNA Experiments*-Small interfering RNA (siRNA) targeting mouse AhR (si-AhR) and non-targeting siRNA (si-ctrl) were synthesized by Dharmacon. 80 pmol of si-AhR and si-ctrl were transfected into αTN4 cells using Lipofectamine2000 (Invitrogen). Total cell protein was extracted by RIPA buffer (Pierce) after 60 h and AhR, αB-crystallin and β-actin protein levels were estimated by immunoblotting.

### Transfection and Reporter Gene/Luciferase Assay

Cells were grown in 24-well plates to 40–50% confluence and co-transfected with pcDNA3.1/B6AhR, pcDNA/ARNT or pcDNA3.1 (vector) and reporter plasmids using Fugene6 (Roche Applied Science). pCMVβ-gal (Clontech) or pRL-CMV (Promega) was co-transfected to normalize for transfection efficiency. TCDD (10 nM) or DMSO (0.01%) was added into the culture medium 24 h after transfection and the cells were incubated for another 24 h. Luciferase activities were determined in 20 µl of cell lysate by a Dual Luciferase Assay kit (Promega) on a GloMax™ 96 microplate luminometer (Promega). Relative luciferase activities (Relative Luciferase Unit, RLU) were expressed as the ratio of the promoter activities generated by the reporter plasmid to that of the control plasmid. Fold change was recorded as the ratio of RLU from experimental group *versus* control group.

### Primary Muscle-Derived Fibroblast Cell Isolation and Culture

Hindlimb skeletal muscles were removed from age matched adult AhR^−/−^ and AhR^+/+^ mice (kindly provided by Dr. Frank Gonzalez; National Cancer Institute). This study was carried out in strict accordance with the recommendations in the Guide for the Care and Use of Laboratory Animals, National Research Council, Institute of Laboratory Animal Resources, 1996. The Animal Study Protocol (06-560) was approved by the Animal Care and Use Committee of the National Eye Institute, National Institutes of Health. The mice were euthanized by exposure to CO_2_ gas following the American Veterinary Medical Association Guidelines on Euthanasia, June 2007 and all efforts were made to minimize suffering. After removing fascia, fat, and connective tissue, the muscles were washed three times in PBS, minced into small pieces and digested with 0.2% trypsin-EDTA (Invitrogen) at 37°C for 30 min before centrifugation at 2,000 rpm for 5 min. The pellets were resuspended in DMEM and incubated at 37°C and 5% CO_2_ for 30 min. Non-adherent cells were removed and the adherent cells were washed with PBS to discard remaining myogenic cells. The fibroblasts were cultured in DMEM medium with 10% fetal bovine serum for several generations.

### Protein and mRNA Preparation

Mice were euthanized by CO_2_ and the cornea, lens, retina, heart, and limb skeletal muscles were surgically excised. Tissue pellet were resuspended in RIPA buffer (Pierce) supplemented with protease and phosphatase inhibitor cocktail (Pierce), homogenized for 30 sec on ice and centrifuged at 14,000×g for 15 min at 4°C. Aliquots of the supernatant fractions were stored at −80°C. For protein preparation, cells were washed by PBS, scrapped and collected. Total protein was extracted at 4°C in RIPA buffer supplemented with protease and phosphatase inhibitor cocktail. The cell lysates were centrifuged and aliquots of the supernatant fractions stored at −80°C. Cell nuclear extracts were prepared using the nuclear extract kit (Active Motif) following the instructions of the manufacturer. For mRNA preparations, the specified tissues were snap frozen in liquid nitrogen and stored at −80°C. mRNA extractions were performed using a Qiagen RNeasy Plus Mini Kit (Qiagen).

### Western blot

Protein lysates were quantified using a BCA Protein Assay kit (Pierce). Equal amounts of protein were subjected to 4–12% Bis-Tris SDS-PAGE (Invitrogen) and transferred electrophoretically onto a nitrocellulose membrane. The membrane was blocked with 5% fat-free milk/PBST, sequentially incubated with primary antibody overnight at 4°C and secondary antibodies for 1 h at room temperature, and autoradiographed by the ECL detection kit (Pierce).

### In Vitro Transcription and Translation

1 µg pcDNA3.1/B6AhR and 1 µg pcDNA/ARNT were separately transcribed and translated *in vitro* using a rabbit reticulocyte lysate expression kit (Promega) following the manufacturer's instruction. The reaction mixtures were combined and incubated with 100 nM TCDD or DMSO (0.01%) for 1 h at 30°C. 5 µl of each lysate was used for gel shift experiments.

### Gel Shift

10 µg nuclear protein or 5 µl *in vitro*-translated protein were mixed with 1 µl poly (dI-dC) in 10 µl buffer (25 mM Hepes, 60 mM KCl, 1 mM DTT, 1% glycerol, 2% CHAPs, 4 mM spermidine) on ice for 30 min. 1 µl ^32^P-labeled oligonucleotides was incubated in the reaction mixture for 30 min at room temperature. For competition experiments, a 25- to 100-fold molar excess of unlabeled oligonucleotides was incubated with the nuclear extract for 30 min on ice prior to addition of the ^32^P-labeled probe. Supershift tests were conducted by incubation of 1 µg AhR or ARNT antibody with nuclear extract prior to the addition of the ^32^P-labeled probe. Protein-DNA complexes were analyzed in a 6% polyacrylamide DNA retardation gel (Invitrogen) using 0.5× Tris borate EDTA (TBE) buffer. The gel was dried and subjected to autoradiography.

### Chromatin Immunoprecipitation (ChIP) Assays

Cells were rinsed with PBS and crosslinked with 1% formaldehyde for 10 min at room temperature; the reaction was stopped by addition of 0.125 M glycine. Cells were rinsed and collected in ice-cold PBS and centrifuged at 3,000 rpm for 5 min at 4°C. The cell pellets were resuspended in 1 ml lysis buffer (50 mM Tris-HCl, pH 8.0, 150 mM NaCl, 5 mM MgCl, 1 mM EGTA, 0.5% TritonX-100, protease inhibitor cocktail freshly added) and incubated on ice for 10 min. The cell lysates were centrifuged at 5,000 rpm for 5 min and nuclei were resuspended in 300 µl nuclear lysis buffer (1% SDS, 50 mM Tris-HCl, pH 8.0, 10 mM EDTA, protease inhibitor cocktail freshly added). The suspensions were sonicated to produce DNA fragments of 300–900 bp, centrifuged at 13,000 rpm for 10 min, and the supernatant fractions removed. The supernatant fractions were pre-blocked with protein G sepharose beads in nine volumes of dilution buffer (lysis buffer) for 1 h at 4°C and centrifuged; subsequently, the supernatant fractions were incubated with 2 ug goat anti-AhR antibody overnight at 4°C with gentle rotation. The immunocomplexes were precipitated by protein G sepharose beads, pelleted and washed sequentially with buffer I (0.1% SDS, 1% TritonX-100, 2 mM EDTA, 20 mM Tris, pH 8, 150 mM NaCl), buffer II (0.1% SDS, 1% TritonX-100, 2 mM EDTA, 20 mM Tris, ph 8, 500 mM NaCl), buffer III ( 0.25 M LiCl, 1% NP-40, 1% NaDOC, 1 mM EDTA, 10 mM Tris, pH 8), and three times with TE buffer. The samples were boiled, centrifuged and the supernatant fractions collected. After dilution with three volumes of water, 2 µl sample were subjected to 50 PCR cycles or processed by real time PCR on a 7900HT Fast Real-Time PCR System (Applied Biosystem) using SYBR® Green PCR Master Mix kit (Applied Biosystem). The primers for amplifying the −265 to −466 fragment of the *αB-crystallin* enhancer containing the XRE-like motif were: 5′-AGC TCA TTC CAG TCA GA and 5′-GCT AGG ATG GAG CCT GGA AT.
